# The ecological and evolutionary energetics of hunter‐gatherer residential mobility

**DOI:** 10.1002/evan.21485

**Published:** 2016-06-17

**Authors:** Marcus J. Hamilton, José Lobo, Eric Rupley, Hyejin Youn, Geoffrey B. West

**Keywords:** spatial ecology, metabolic theory of ecology, body size, temperature‐dependence, net primary production

## Abstract

Residential mobility is a key aspect of hunter‐gatherer foraging economies and therefore is an issue of central importance in hunter‐gatherer studies.^1^, ^2^, ^3^, ^4^, ^5^, ^6^, ^7^ Hunter‐gatherers vary widely in annual rates of residential mobility. Understanding the sources of this variation has long been of interest to anthropologists and archeologists. The vast majority of hunter‐gatherers who are dependent on terrestrial plants and animals move camp multiple times a year because local foraging patches become depleted and food, material, and social resources are heterogeneously distributed through time and space. In some environments, particularly along coasts, where resources are abundant and predictable, hunter‐gatherers often become effectively sedentary. But even in these special cases, a central question is how these societies have maintained viable foraging economies while reducing residential mobility to near zero.

While the causes of hunter‐gatherer mobility undoubtedly include a combination of cultural, economic, and biological factors, we focus here on the coarse‐grained ecological and energetic constraints of residential mobility. We define hunter‐gatherer residential mobility as the pattern of camp‐to‐camp movements of individuals within a population over the course of a year to exploit their environments effectively to meet their nutritional, material, and social requirements. Residential mobility is commonly measured as the total distance moved per year and the number of moves made per year. An important metric of interest is thus the average distance per residential move, particularly as mobility decisions are commonly made on a short‐term basis rather than annually.

Although residential mobility is fundamental to the hunter‐gatherer way of life, it is extremely costly in terms of energy (the basic metabolic cost of movement), logistics (the successful planning and organization of moving individuals, families, and their material culture), opportunity loss (time is spent moving camps rather than foraging, child‐rearing, producing tools, or any of the many other tasks contributing to fitness), and time (there is a finite number of days in the year). Given these costs, residential mobility constitutes a large fraction of a hunter‐gatherer energy budget (or opportunity, logistic, or time budget). It is therefore reasonable to assume that these costs should be minimized within the constraints of local ecosystems.

How predictable is variation in hunter‐gatherer residential mobility? We address this central question using ethnographic data, with which we assess a set of hypotheses derived from formal theory^8^, ^9^, ^10^; these hypotheses make specific predictions about the scale and variation of hunter‐gatherer residential mobility. These predictions are based on the evolved biomechanics and bioenergetics of humans, and the turnover of energy in ecosystems. In addition, we address the implicit question of whether hunter‐gatherer mobility is predictable from what is known of evolutionary and ecological constraints on mammalian mobility.

Our framework jointly considers two activities that are central to hunter‐gatherer spatial ecology: use of space, where space is taken to be the resource catchment area for food, water, and all material and social resources needed for survival, and movement in space, or the set of constraints that shape the effective use of space and the energetic limits of body size. Our goals are to link the use of space and movement in it in a formal conceptual framework and to show empirical support for the predictions generated by such a framework using independent data sets on hunter‐gatherer area use and mobility. Our model explains and predicts the variation in observed residential mobility. These results can be extended both to the past and the present for cases where data are not currently available. Specifically, we show that both the scale of hunter‐gatherer mobility and variation in rates of mobility across different environments can be mathematically derived from fundamental ecological theory. We present a conceptual flow chart of our theoretical approach in Figure 1.

**Figure 1:**
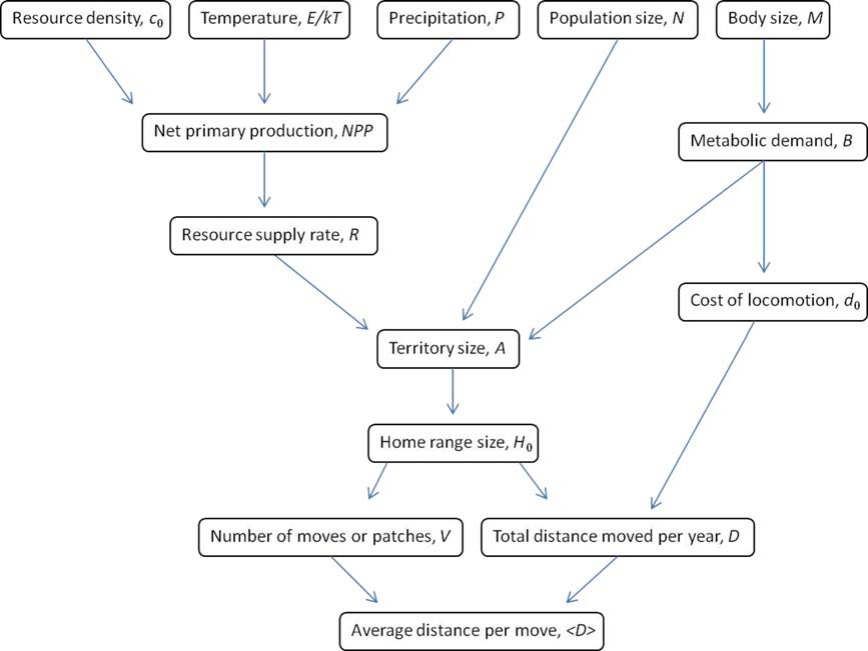
Flow chart of environmental, population and individual level processes influencing hunter‐gatherer use of space and residential mobility examined in this paper. [Color figure can be viewed in the online issue, which is available at wileyonlinelibrary.com.]

## THEORETICAL FRAMEWORK

Since mobility is, to a large extent, driven by the need for a continuous supply of energy in the form of food resources, a natural framework for addressing this question is provided by the metabolic theory of ecology.^8^, ^10^ This body of theory provides a powerful framework for formulating formal, testable hypotheses concerning evolutionary and ecological constraints on the scale and variation of hunter‐gatherer residential mobility. It is based on the observation that energy supply and use are fundamental to all biological and ecological processes and that “metabolism is to ecology as genetics is to evolution.”^8^ Understanding how energy fluxes through organisms (that is, metabolism), and how this scales across units of biological organization (which are essentially collections of organisms, such as populations, communities, ecosystems, and the biosphere) is as fundamental to understanding ecology as is understanding how genetic processes operating at the same scales of biological organization are fundamental to evolution.

The metabolic theory of ecology is based on the empirical observation (known as Kleiber's Law) that the basal metabolic rate, *B*, of an organism scales with its mass, *M*, as a simple power law:

(1) $$B=B_{0}M^{\alpha }$$

where $B_{0}$ is a taxon‐specific constant and α≈3/4. Similar scaling laws hold for almost all physiological traits and life‐history events across all taxonomic groups, manifesting exponents that are typically simple multiples of ¼. The origin of these ubiquitous 1/4‐power scaling laws arises from the fractal‐like, space‐filling nature of optimized internal networks, such as the vascular system, that distribute the energy and resources that sustain organisms.^11^, ^12^, ^13^ The overall scale parameter, $B_{0}$, is derived from the underlying biochemical kinetics of metabolism and, accordingly, depends on the temperature, *T*, at which an organism operates (14). This is given by the exponential Arrhenius‐Boltzmann factor, exp(−E/kT), where *E* (∼0.6 eV) is the average activation energy of the biochemical reactions contributing to metabolic processes, *k* is Boltzmann's constant ( $8.62\times 10^{-5}$ eV), and *T* is the absolute temperature at which the organism operates (^o^K). Incorporating this into equation (1) yields^14^

(2) $$B=B_{1}e^{-E/kT}M^{\alpha }$$

where $B_{1}$ is a mass‐ and temperature‐independent constant. For endotherms, *T* is the internal body temperature; for ectotherms, *T* is the ambient environmental temperature. A large body of empirical work supports equation (2) as a fundamental description of the metabolic rate of individual plants and animals. Essentially, it states that the metabolic rate of an organism is a function of its size, the structure of its internal distribution networks, and the temperature at which it operates. Put slightly differently, it expresses the fact that mass and temperature are the major determinants of the variation of physiological traits and life‐history events, and that these are encapsulated in just two numbers, ¼, derived from network dynamics, and E ∼ 0.6 eV, the scale of fundamental biochemical reactions.

Since ecosystems are composed of individual organisms, it follows that the respiration (or metabolism) of an entire ecosystem, *R*, is the sum over all individuals, $B_{i}$:^15^

(3) $$R=\sum_{i}B_{i}$$

where *i* indexes individuals. Because the vast majority of biomass in an ecosystem is composed of microbes and plants, both of which are ectothermic, it follows that the temperature‐dependence of the metabolic rate of an ecosystem is primarily governed by a single Arrhenius‐Boltzmann factor:

(4) $$R\propto e^{-E/kT}$$

where *T* is the ambient environmental temperature. Importantly, because equation (4) describes the temperature‐dependent metabolic flux of an ecosystem, all biotic interaction rates associated with metabolism, such as biomass growth, disease load, and predator‐prey interaction rates, are predicted to exhibit the same temperature‐dependence.^8^

In addition to environmental temperature, there are other important rate‐limiting constraints on *R*, such as the availability of water.^8^, ^9^ For example, not all warm ecosystems have high rates of biomass turnover (for example, warm deserts), whereas some do (for example the tropics). Therefore, we introduce annual precipitation, *P* (mm/yr), as the rate‐limiting constraint into equation (4), giving the expression

(5) $$R\propto P^{\theta }e^{-E/kT}$$

where the exponent θ captures the response of a change in resource supply rate to a change in precipitation.

In the rest of this paper, we use equation (1) to predict the overall scale of hunter‐gatherer residential mobility based on human body size and equation (5) to predict the variation in rates of residential mobility across hunter‐gatherer cultures based on ecosystem energetics.

## DATA

We use three data sets. The first is terrestrial mammal migration data from Hein, Hou, and Gillooly^16^ The data is in terms of two variables (*n* = 33): Mammalian body size (*M*), or the average body size of a species in kg and annual migration distance (*D*), which is the total migration distance covered by an individual of that species per year in km. The sample size is relatively small due to the paucity of high‐quality data. The second data set is a collection of estimates (n = 47) of the total distance traveled per year (*D*) by hunter‐gatherer groups, from Kelly.^1^

The third data set is hunter‐gatherer spatial ecology using data from Binford.^3^ The data we use consist of seven variables and include all groups that move at least once per year (*n* = 314):

1. Area (*A*): the total territory size used by a population of hunter‐gatherers over a year.
2. Population size (*N*): the total population size in an ethnolinguistic group.
3. Distance traveled per year (*D*): the total distance moved over the course of the year during residential movements from patch to patch (in km).
4. Number of moves (*V*): the total number of residential moves made in a year.
5. Average annual temperature (*T*): the average environmental temperature of the population.
6. Precipitation (*P*): the average annual rainfall experienced by the population (in mm/yr).
7. Net primary production (*NPP*): the net production of biomass produced over the course of a year in an ecosystem (in g/m^2^/yr; that is, the annual turnover of biomass. *NPP* is therefore a measure of the flux of free energy in an ecosystem.

The Binford data is the largest and most recent cross‐cultural collection of data on global variation in hunter‐gatherer ecology currently available. The data were collated primarily from primary sources in the ethnographic and ethnohistoric literature. Thus, like any dataset, this one is subject to errors introduced during collection and collation. However, assuming that data assembly was not systematically and simultaneously biased across multiple variables, the sources of error should be statistically independent. The predominant effect should therefore be to introduce noise into the analysis, without significantly affecting underlying statistical trends. The Kelly data set is similar to the Binford data, collected in similar ways, and so is likely subject to the same sources of error.

## EVOLUTIONARY CONSTRAINTS ON THE SCALE OF HUNTER‐GATHERER MOBILITY

Because food resources are heterogeneously distributed in time and space, and energy storage capacity is strictly limited, mobility is a fundamental component of the ecology of most animals.^17^, ^18^ This, of course, is the case for most mobile hunter‐gatherers: Their local resource patches become depleted over time, and the capacity to store food in most environments is limited to no more than a few days without effective bulk‐food preservation techniques. While traditional storage technologies such as drying or smoking meat are (and were) widely used, their effectiveness is limited. The preserved meat is rarely able to support the total energy requirements of local populations for any substantial amount of time except in a very limited set of circumstances. For example, hunter‐gatherers can become effectively sedentary or semi‐sedentary in locally specific environments, especially along coasts or lake shores, where resources are abundant, predictable, and storable. However, this is rarely the case for terrestrial foragers, the vast majority of which are nomadic and residentially mobile.

Because mobility is energetically costly, it is always minimized, given the biomechanical and bioenergetic constraints of an organism.^16^, ^17^, ^18^ This minimization principle is well‐known in mammals, which primarily migrate by walking, where total annual migration distance, *D*, scales with body size *M*, as^16^

Because mobility is energetically costly, it is always minimized, given the biomechanical and bioenergetic constraints of an organism.

(6) $$D=d_{0}M^{1/3}$$

where $d_{0}$ is a constant capturing the ratio of the capacity of mammals to store energy to the metabolic cost of transport, both of which scale with body size. The 1/3‐scaling with body size results from the geometric principle that stride length is a linear dimension, and so scales as a 1/3‐power of a volume (that is, body size).

Given that humans are mammals, mobile hunter‐gatherers have extremely limited abilities to store significant amounts of energy beyond body fat, and local resources become depleted requiring residential movements to new patches,^19^ we hypothesize that equation (6) describes the average total distance moved per year across our sample of hunter‐gatherer societies. Specifically, we test the hypothesis that the total distance moved per year by the average terrestrial hunter‐gatherer group is consistent with that predicted by body size. We examine this using mammal body size and migration distance, as described earlier, against which we plot the average (±2 s.d.'s) total distance moved per year as a function of average hunter‐gatherer body size.

**Figure 2:**
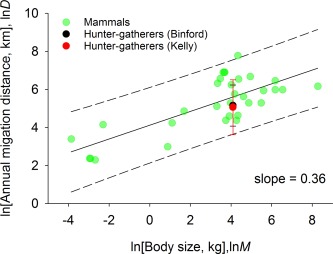
Annual migration distance as a function of body mass for terrestrial mammals and hunter‐gatherers on logarithmic axes. Hunter‐gatherer estimates are mean +/− 1 s.d. (Binford: 158.43 +/− 2.94; Kelly: 174.08 +/− 4.22 km/yr).

Figure 2 shows that, using both the Kelly and the Binford data sets, the total distance moved per year by hunter‐gatherers is remarkably close to the expected annual migration distance for a 60‐kg mammal. This demonstrates that the overall scale of mobility exhibited across hunter‐gatherer societies is a function of the evolutionary biomechanics and bioenergetics of human body size. While human body size sets the overall scale of hunter‐gatherer annual mobility, we now turn to understanding variation in rates of residential mobility observed across hunter‐gatherer populations.

## ECOLOGICAL CONSTRAINTS ON THE VARIATION OF HUNTER‐GATHERER MOBILITY

Over human evolutionary history, hunter‐gatherers have existed in the vast majority of terrestrial environments across the planet.^1^ Hamilton and coworkers^20^ showed that the size of a hunter‐gatherer territory varies predictably with coarse‐grained environmental constraints, specifically environmental temperature and water availability. This is important because the total size of a territory is the sum of individual home ranges, *H*, which are defined as the area used by an individual of body size, *M*, to meet its metabolic requirements, *B*, given the resource supply rate (or energy availability) per unit area, *R*, of the local environment. Effectively, a home range is a resource catchment area. Home range is thus defined as $H_{0}\equiv B/R$ and a territory size, *A*, consisting of *N* individuals is

(7) $$A=\sum_{i}^{N}H_{0,i}$$

Because humans do not differ in body sizes by orders of magnitude, we hold *M*, and consequently *B,* fixed, corresponding to an average mass of 60 kg (see Walker and Hamilton^21^ for further discussion of variation in hunter‐gatherer body sizes). Hamilton and coworkers^20^ also showed that hunter‐gatherers exhibit economies of scale in their spatial ecology, reflected in the area per individual, *A/N*, decreasing predictably with population size. Here, the term “economies of scale” refers to the cost or efficiency advantages accrued with increased output or level of activity. Because $A=H_{0}N^{\beta }$, where β≈3/4, the area per individual scales as^20^

(8) $$A/N=H_{0}N^{-1/4}$$

Equation (8) captures an economy of scale because it quantifies the energetic benefits to an individual existing within a population of *N* individuals. Assuming that home ranges are constant, body size is not density dependent,^18^ and there is no significant improvement in extractive technologies, equation (8) implies that home ranges effectively overlap with larger populations *N*. This results in an effective reduction in the exclusive area an individual requires to meet his or her metabolic requirement, *A/N*, at a rate proportional to $N^{-1/4}$.^20^, ^22^

**Figure 3:**
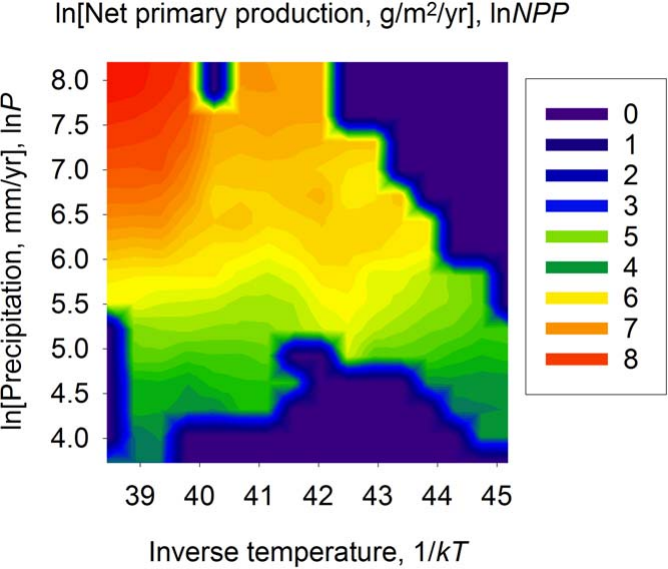
Net primary production as a function of inverse temperature and precipitation. High‐ intensity reds indicate highest *NPP*; low‐intensity greens or blues indicate low *NPP*.

As stated earlier, variation in hunter‐gatherer space use is constrained by the average environmental temperature and water availability within territories. Assuming nutrient availability,^23^ temperature and water availability yields a net primary production (*NPP*), which can be defined as the density of biomass produced per year (g/m^2^/yr). Because *NPP* is measured in units of time and area, it is the annual flux of useful chemical energy (Gibbs free energy) in an ecosystem that is used to produce new biomass. Indeed, a multiple regression of ln NPP=f(1/kT, ln P) from hunter‐gatherer territories demonstrates that 83% of the variation in *NPP* is explained by these two variables (see Table 1 for regression results). The contour plot in Figure 3 shows that *NPP* is highest in warm, wet hunter‐gatherer territories (the tropics) and lowest in cold, dry environments (arctic or tundra). This is important for hunter‐gatherer ecology because biodiversity increases monotonically with *NPP*.^24^, ^25^ Therefore, so does the potential diversity of harvestable plants and animals.^1^

**Table 1:** Regression Statistics and Coefficients for Net Primary Production ln*NPP*) as a Function of Temperature 1/*kT*) and Precipitation ln*P*)

ANOVA table					
Source	DF	Adj SS	Adj MS	F‐Value	P‐Value
Regression	2	349.47	174.74	818.84	0.000
*E*	1	18.00	18.002	84.36	0.000
ln*P*	1	196.35	196.35	920.12	0.000
Error	336	71.70	0.21		
Total	338	421.17			
R‐sq	R‐sqadj)				
82.98%	82.87%				

Coefficients				
Term	Coef	SE Coef	T‐Value	P‐Value
Constant	6.841	0.84	8.13	0.000
*E*	−0.16	0.02	−9.18	0.000
ln*P*	0.95	0.03	30.33	0.000

In the following, however, we are interested in measuring the individual effects of temperature and water availability on hunter‐gatherer spatial ecology and so treat the two components of *NPP* individually. As such, the environmental constraints on hunter‐gatherer home range are described by:

(9) $$H_{0}=\frac{\left\langle B\right\rangle }{R}=\frac{\left\langle B\right\rangle }{c_{0}P^{\theta }e^{-E/kT}}$$

where $c_{0}$ is a constant. We then write a full expression of the scaling of hunter‐gatherer territory sizes as

(10) $$A=\langle B\rangle c_{0}^{-1}P^{-\theta }e^{E/kT}N^{\beta }$$

The area per individual is therefore given by

(11) $$A/N\propto P^{-\theta }e^{E/kT}N^{\beta -1}$$

The above expression describes the environmental‐ and population size‐dependence of the area required by an individual to meet his or her annual metabolic requirements. Having established the environmental constraints on individual space‐use by hunter‐gatherers,^20^, ^22^ we now turn to modeling the mobility strategy employed to access that space.

By definition, mobile hunter‐gatherers divide the total area they require to meet their annual energy budget into a discrete number of annual moves between patches. Under the assumption that hunter‐gatherers will minimize the energetic costs of accessing this area, we hypothesize that, on average, rates of hunter‐gatherer mobility should show the same temperature dependence as the area use described earlier. That is to say, two fundamentally interdependent aspects of hunter‐gatherer spatial ecology are home‐range size and mobility and, accordingly, both should show the same environmental dependence.

**Figure 4:**
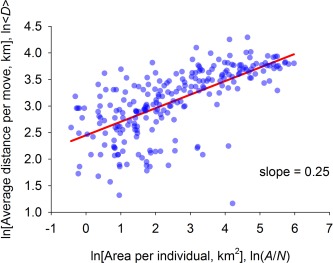
Average distance per move as a function of area per individual on logarithmic axes (OLS regression: *r^2^* = 0.43, *p*<0.0001, *d.f*.=238).

A mobile hunter‐gatherer population moves *V* times per year, covering a total distance *D* over a year. Therefore, the average distance per move (that is, distance between residential patches) is ⟨D⟩≡D/V. Given equation (11), we hypothesize that ⟨D⟩=f(T,P,N). Figure 4 shows that

(12) $$\langle D\rangle \propto {\left(\frac{A}{N}\right)}^{1/4}$$

Combining equations (11) and (12) leads to the prediction that

(13) $$\left\langle D\right\rangle \propto P^{-\frac{1}{4}}e^{\frac{E}{4}\frac{1}{kT}}N^{-\frac{1}{16}}$$

**Figure 5:**
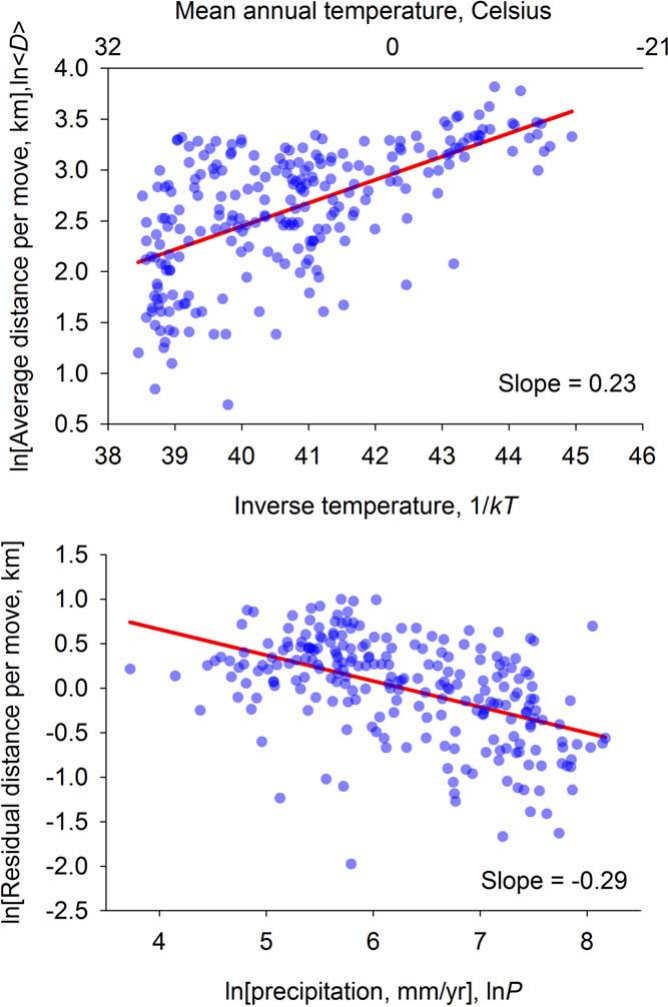
Average distance per move as a function of temperature and precipitation, on semi‐log axes. Top panel: Distance and temperature, with slope OLS regression: *r^2^*=0.36, *p*<0.0001, *d.f*.=238). Bottom panel: Residual distance and precipitation OLS regression: *r^2^*=0.24, *p*<0.0001, *d.f*.=238).

Because the scaling of ⟨D⟩ with *N* is so shallow (with a scaling exponent of only −1/16), for small hunter‐gatherer populations its value is effectively zero. We test this prediction using the hunter‐gatherer annual mobility data from Binford.^3^ Consistent with our predictions, Figure 5 shows that the average distance per move is both temperature‐ and precipitation‐ dependent. More accurate estimates of the scaling exponents result from a regression model that captures the simultaneous effects of temperature, rainfall, and population size on average travel distance (see Table 2 for results). Again, consistent with our predictions (equation (14)), results show that

(14) $$\left\langle D\right\rangle \propto P^{-0.27}e^{0.15\frac{1}{kT}}N^{0.01}$$

Therefore, not only is average distance per move temperature‐dependent, but at the same rate as area use, and this has been shown using independent datasets.

**Table 2:** Regression Statistics and Coefficients for Average Distance per Move ln ⟨D⟩ as a Function of Temperature (*E*), Precipitation (ln*P*), and Population Size (ln*N*)

ANOVA table					
Source	DF	Adj SS	Adj MS	F‐Value	P‐Value
Regression	3	44.98	14.99	73.89	0.000
*E*	1	9.47	9.47	46.69	0.000
ln*P*	1	11.21	11.21	55.25	0.000
lnN	1	0.06	0.06	0.29	0.593
Error	234	47.48	0.20		
Total	237	92.46			
R‐sq	R‐sqadj)				
48.65%	47.99%				

Coefficients				
Term	Coef	SE Coef	T‐Value	P‐Value
Constant	−1.72	0.983	−1.76	0.080
*E*	0.15	0.02	6.83	0.000
ln*P*	−0.27	0.04	−7.43	0.000
ln*N*	0.01	0.02	0.54	0.593

## DISCUSSION

In this paper, we have shown that the average distance a hunter‐gatherer band moves between patches is a direct function of the available energy in the local environment. Moreover, and importantly, we have shown that it is possible to quantitatively predict how hunter‐gatherer mobility should vary across different environments, given the fundamental biochemistry and kinetics of energy turnover in ecosystems. As predicted by ecological theory, the average distance between patches increases exponentially with decreasing temperature and decreasing precipitation in response to decreasing flux of energy in ecosystems.

Specifically, we considered two hypotheses concerning hunter‐gatherer rates of residential mobility derived from the metabolic theory of ecology. The first states that, as a consequence of biomechanical, bioenergetic, and geometric constraints, the overall scale of hunter‐gatherer annual residential mobility is determined by our species body size. Ethnographic data suggest that, indeed, average levels of hunter‐gatherer annual mobility are close to the predicted level of mobility for a 60‐kg mammal, given the limited capacity to store energy. The second set of predictions state that since hunter‐gatherer rates of mobility are responsive to energy availability in ecosystems, they are dependent on water availability and the flux of energy in ecosystems. These rates of mobility are predicted by metabolic theory and are well supported by available data. We show that the overall scale of hunter‐gatherer mobility is set by human evolutionary biomechanics and bioenergetics, and that the observed variation in the rates of hunter‐gatherer residential mobility across cultures is largely a function of energy availability in ecosystems.

We show that the overall scale of hunter‐gatherer mobility is set by human evolutionary biomechanics and bioenergetics, and that the observed variation in the rates of hunter‐gatherer residential mobility across cultures is largely a function of energy availability in ecosystems.

Given the well‐known positive temperature‐dependence of biodiversity, measured as the abundance of species per unit area, including plants, mammals, reptiles, amphibians, and birds,^8^, ^10^, ^24^, ^25^ our results suggest that hunter‐gatherer mobility decreases as the potential diversity of prey species increases. Moreover, our results suggest that because biotic interaction rates are temperature‐dependent, foraging interaction rates with prey species also increase with temperature and precipitation. That is to say, in warmer and wetter environments there will be a predictably higher diversity of potential prey species (see Hatton and colleagues^26^) and, therefore, a higher interaction rate with harvestable resources.

However, temperature also has negative impacts on hunter‐gatherer mobility. The same theory also predicts that because human pathogen load increases predictably with ecosystem temperature, warmer, and especially wetter environments, will have a greater disease interaction rate.^27^ Combined with pathogen load and the fact that decay rates will also increase predictably with increasing temperature, limiting the ability to store food resources, disease avoidance and storage capabilities will increase rates of residential mobility in warm and wet environments. Indeed, in our dataset, the eight groups that reported the highest number of moves per year are all tropical foragers. (In some cases, these estimates may be considerable underestimates; this is true, for example, of the Ache^28^ and the Nukak.^29^)

**Figure 6:**
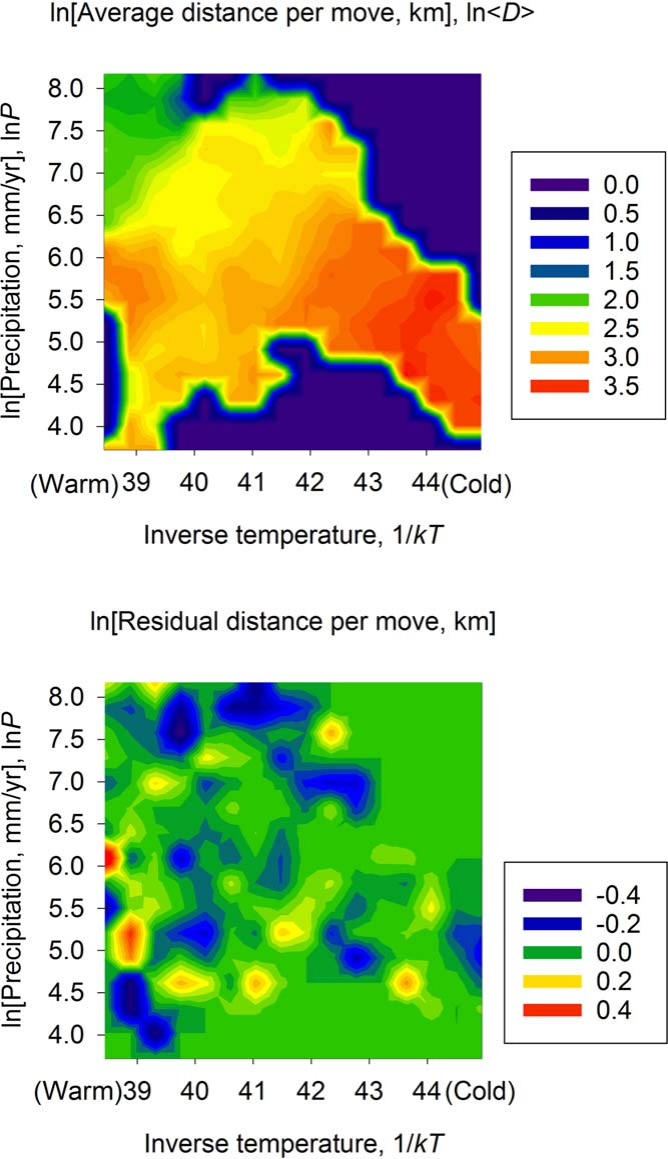
Contour plots of the average distance per move (top) and the residuals (bottom) as a function of annual precipitation and inverse temperature.

One way to visualize the remarkable empirical congruence between residential mobility and the availability of energy in ecosystems is to compare the contour plots of net primary production, Figure 3, and average distance per move, Figure 6. These plots show that in two dimensions, temperature and precipitation, there is a remarkable similarity between *NPP* and averaged distance moved. Average distance moved is highest in regions of lowest *NPP* and decreases as *NPP* increases along very similar two‐dimensional gradients. These plots confirm that rates of hunter‐gatherer mobility track gradients in the flux of energy in ecosystems. The bottom panel of Figure 6 shows that there is no clear structure to the residuals of average distance, indicating that the relationships among mobility, temperature, and precipitation are equally predictive across the two‐dimensional plot.

The results shown here are consistent with data and analysis reported in Kelly,^1^ where the average distance per move by hunter‐gatherer groups decreases exponentially with effective temperature. However, because Kelly reports temperature as “effective temperature,” these data cannot be used to validate our theory directly. Our results are also consistent with Binford's^3^ “packing model,”, in which hunter‐gatherer mobility decreases with population density (holding environmental variation constant). For example, rearranging equation (12), we find $\langle D\rangle \propto {\left(N/A\right)}^{-1/4}$, which states that the average distance per move decreases with population density to one quarter‐power. This is particularly interesting because the naïve geometric expectation would be that a distance traveled should scale as the square root of the area covered (that is, $L\propto A^{1/2}$). However, the ¼‐power scaling relation between ⟨D⟩ and A exhibited in the data (Fig. 4 and equation (12)) indicates an unexpected economy of scale in residential mobility, similar to that shown for area use (equation (8)). Therefore, this observation suggests a further mechanistic connection between hunter‐gatherer use of space and mobility in which the economy of scale in spatial energy use may result from the economy of scale in the mobility strategy used to access that space.

It is also interesting to note that from our results (equation (14)), the average distance per move is effectively independent of population size (that is, $\langle D\rangle \propto N^{0}$). This has two important implications. One is that the density‐dependence of mobility is not a function of the number of people in the overall population, but in the amount of area they require to meet their requirements. The other is that in those cases in prehistory in which terrestrial hunter‐gatherers became sedentary before the full development of agriculture, such as the Natufian Near East,^30^ the primary cause of sedentism was probably not increasing population sizes *per se,* but changes in their subsistence ecology (effectiveness at utilizing the spatial availability of energy).

While the hunter‐gatherer dataset used here is both large and geographically extensive, it is, by construction, exclusively historic in nature, limited to those hunter‐gatherer societies for which written records exist in either the present or recent past (that is, the “ethnographic present”). Therefore, it is reasonable to be concerned that this is a biased sample of hunting and gathering as a human life style *in toto,* since we have fragmentary archeological evidence of either prehistoric or preagricultural hunter‐gatherers. However, the strength of the explanatory approach in this paper, as opposed to ethnographic analogy,^31^ is that by understanding how underlying dynamics derived from general principles constrain observed distributions, we are able to make robust inferences about societies for which we have little or no data. Moreover, there is no *a priori* reason to believe that rates of residential mobility and area use in prehistoric or preagricultural hunter‐gatherer societies would not have responded to such fundamental ecological constraints as the availability of energy on landscapes, which we see in ethnohistoric data. Indeed, the contour map, Figure 6, provides predictions for the average distance an individual in a hunter‐gatherer population could be expected to move, given any combination of environmental temperature and annual precipitation, irrespective of time period. Thus the model provides novel quantitative insights into the conditions under which terrestrial hunter‐gatherers may become sedentary. The predictive nature of this approach to understanding major transitions in human energetics will be the subject of future work.

Our results provide a statistically robust mechanistic explanation of how large‐scale ecological constraints fundamentally shape the movement of hunter‐gatherers through space as they use available energy. Importantly, however, there is unexplained variation in our models. Sources of this unexplained variation likely include cultural, economic, and ecological processes, historical contingency, and error in measurements and statistics. Therefore, our claim is not that all aspects of hunter‐gatherer residential mobility (and spatial ecology as a whole) are simply responses to energy availability: The ways in which cultural mechanisms affect hunter‐gatherer spatial ecology are of great interest, but are much harder to measure and are not explicitly considered here. Nevertheless, the overall constraints that shape hunter‐gatherer spatial ecology are regular, predictable, and coarse‐grained aspects of the environment.
